# Creosotal in Pneumonia

**Published:** 1906-06

**Authors:** 


### ABSTRACTS.

---

### Creosotal in Pneumonia.

Prof. Andrew H. Smith, of New York, (*Am. Therapist,* January 15, 1905) says that in discussions of pneumonia, the pneumococcus is often confused with the pneumotoxin. The first is a microorganism which finds a nidus in the aircells of the lungs, constituting a local lesion; the second is generated by the first and then absorbed into the blood, giving rise to acute fever. Perhaps a majority of persons almost constantly carry pneumococci in that part of the airway which is lined with ciliated epithelium, but there the cocci do not multiply freely and are unirritating. So long as the ciliæ remain active they oppose the advance of the germs toward the aircells, where there is a pavement epithelium. But the introduction of a single germ into the congenial nidus afforded by the air vesicle may be sufficient to set up the process; and as the organisms multiply and the toxin from them is absorbed into the blood, the infectious fever appears.

This is prefatory to the question whether it is possible to abort or favorably influence the course of pneumonia by drugs. Are the germs in the lung and their products in the blood capable of being so acted upon by safe doses of antiseptics as to be deprived wholly or partly of their harmful effects? Many affirm, some deny, while perhaps the majority consider the question sub judice. I do not hesitate to range myself with the first class and to own to impatience with the second group. For more than a decade evidence has accumulated to the fact that a number of substances, possessing bactericidal or antiseptic properties when introduced into the system, do influence materially the course of the disease and diminish its severity.

Of these the one in which I have most confidence is creosote carbonate or creosotal. It reverses the usual proportion of crisis termination to lysis termination, so that the former,—ordinarily much the more frequent,—becomes much the less frequent. This fact has been observed by Peabody, W. H. Thomson, myself and others and can scarcely be without significance. The favorable effect of creosotal upon the mortality is testified to by a long list of authorities. The very extensive bibliography is so easily accessible that I will not burden this article with its reproduction.

In a paper contributed to the Medical and Surgical Report of the Presbyterian Hospital for 1904, Drs. G. A. Tuttle and H. S. Carter report 600 pneumonia cases treated in the hospital during the six years ending February 1903. The mortality in uncomplicated cases was 22.8%, in complicated ones 36%, and, when connected with other diseases, 47%. 101 cases, of which thirty were complicated, received ten minims of creosotal every two hours during the whole course of the disease, in addition to the usual sympathetic treatment. According to the ratio in the other cases, the thirty complicated cases should have given eleven deaths and the seventy-one uncomplicated ones sixteen deaths,—together twenty-seven deaths. The actual number of fatalities was sixteen,—nine in complicated and seven in uncomplicated cases. The mortality in the uncomplicated cases under creosotal was therefore 7%, as against 22.8% in uncomplicated cases under other treatment.

In most of the cases reported in the United States the dose of creosotal rarely if ever exceeded ten minims every two hours. My friend, Dr. W. W. Baldwin of Rome, Italy, reports to me, however, that he gives as much as thirty or forty minims every three hours and has treated eighteen consecutive cases without a single death, even when the prevailing type of this disease was fatal. He never saw any disagreeable symptoms from its use. I personally have never ordered more than fifteen minims every two hours, but should not hesitate to do so if that dose did not reduce the temperature within twenty-four hours. I know of no instance in which harmful effects were noted. Smoky urine appears, but is of no moment and negligible.

It is important to begin the treatment at an early stage of the disease, so that the first exudate poured into the aircells may be impregnated with the drug and rendered an unfavorable culture medium for the germs. When once the cell is packed full with fibrin the plug will be but little affected by any remedial agent in the blood. But the early institution of the treatment is usually precluded in hospital and consultation practice. In family practice there is a natural hesitancy to use an unfamiliar treatment. Therefore the conditions are seldom favorable for the fullest success of creosotal; nor will this be otherwise so long as the efficiency of any specific is dogmatically denied.

Creosotal has incidentally a decided effect in preventing the formation of gas in stomach and intestines, with all the discomfort and danger attending abdominal distension. Should I myself be attacked by pneumonia, I should wish to be treated by a physician who believes in creosotal and will employ it with a liberal hand.

